# Determinants of Deliberate COVID-19 Spread: Exploring Psychological, Social, and Environmental Factors Behind Virus-Spreading Behaviors During a Pandemic in Saudi Arabia

**DOI:** 10.1192/j.eurpsy.2025.1280

**Published:** 2025-08-26

**Authors:** S. Alsubaie, H. Alshahrani

**Affiliations:** 1 Psychiatry, AFHSR, Khamees Mushait; 2 Psychiatry, SCHS, Abha, Saudi Arabia

## Abstract

**Introduction:**

While most individuals adhere to preventive measures during pandemics, a subset engages in behaviors that may intentionally spread COVID-19, presenting unique challenges to public health efforts.

**Objectives:**

This study explores the psychological, social, and environmental factors influencing deliberate virus-spreading behavior in the Saudi Arabian population during the initial phase of the COVID-19 pandemic.

**Methods:**

A cross-sectional online survey was conducted between April 3 and May 5, 2020, with 1,817 participants. The survey collected demographic information and variables related to virus-spreading inclinations. Descriptive statistics and Pearson’s chi-squared tests were used to analyze associations between deliberate virus-spreading behavior and various characteristics, with statistical significance set at p < 0.05.

**Results:**

Individuals who intentionally spread the virus (2% of the sample) were younger (mean age 22.9 vs. 29.3 years, p < 0.001), predominantly female (81.1% vs. 65.6%, p = 0.033), and more likely to have a history of mental health issues (24.3% vs. 8.5%, p < 0.001) and conspiracy beliefs (45.9% vs. 26.2%, p = 0.003) compared to those who did not engage in virus-spreading behaviors. No significant differences were found regarding marital status, education level, or traumatic life events.

**Conclusions:**

This study highlights key factors associated with intentional virus-spreading behaviors, such as younger age, mental health history, and conspiracy beliefs, underscoring the need for integrated mental health support and targeted misinformation interventions. Public health strategies should include age-appropriate messaging and digital outreach to mitigate the risks posed by intentional spread behaviors and enhance pandemic response efforts. Further research with larger, more diverse samples is recommended to expand understanding and improve intervention strategies

**Disclosure of Interest:**

None Declared

